# Association study of *GBA1* variants with MSA based on comprehensive sequence analysis -Pitfalls in short-read sequence analysis depending on the human reference genome-

**DOI:** 10.1038/s10038-024-01266-1

**Published:** 2024-07-18

**Authors:** Kenta Orimo, Jun Mitsui, Takashi Matsukawa, Masaki Tanaka, Junko Nomoto, Hiroyuki Ishiura, Yosuke Omae, Yosuke Kawai, Katsushi Tokunaga, NCBN Controls WGS Consortium, Hatsue Ishibashi-Ueda, Tsutomu Tomita, Michio Noguchi, Ayako Takahashi, Yu-ichi Goto, Sumiko Yoshida, Kotaro Hattori, Ryo Matsumura, Aritoshi Iida, Yutaka Maruoka, Hiroyuki Gatanaga, Akihiko Shimomura, Masaya Sugiyama, Satoshi Suzuki, Kengo Miyo, Yoichi Matsubara, Akihiro Umezawa, Kenichiro Hata, Tadashi Kaname, Kouichi Ozaki, Haruhiko Tokuda, Hiroshi Watanabe, Shumpei Niida, Eisei Noiri, Koji Kitajima, Yosuke Omae, Reiko Miyahara, Hideyuki Shimanuki, Yosuke Kawai, Katsushi Tokunaga, Tatsushi Toda, Shoji Tsuji

**Affiliations:** 1 Department of Neurology, Graduate School of Medicine, The University of Tokyo, 7-3-1 Hongo, Bunkyo-ku, Tokyo 113-8655 Japan; 2 Department of Precision Medicine Neurology, Graduate School of Medicine, The University of Tokyo, 7-3-1 Hongo, Bunkyo-ku, Tokyo 113-8655 Japan; 3 Institute of Medical Genomics, International University of Health and Welfare, 4-3, Kozunomori, Narita-shi, Chiba, 286-8686 Japan; 4 Department of Neurology, Okayama University Graduate School of Medicine, Dentistry and Pharmaceutical Sciences, 2-5-1, Shikata-cho, Kita-ku, Okayama, 700-8558 Japan; 5 Genome Medical Science Project, National Center for Global Health and Medicine, 1-21-1, Toyama, Shinjuku-ku, Tokyo 162-8655 Japan; 6 NCVC Biobank, National Cerebral and Cardiovascular Center, Suita, Osaka, 564-8565 Japan; 7 Medical Genome Center, National Center of Neurology and Psychiatry, Kodaira, Tokyo 187-8551 Japan; 8 Department of Bioresources, Medical Genome Center, National Center of Neurology and Psychiatry, Kodaira, Tokyo 187-8551 Japan; 9 Department of Clinical Genome Analysis, Medical Genome Center, National Center of Neurology and Psychiatry, Kodaira, Tokyo 187-8551 Japan; 10 NCGM Biobank, National Center for Global Health and Medicine, Shinjuku-ku, Tokyo 162-8655 Japan; 11 AIDS Clinical Center, National Center for Global Health and Medicine, Shinjuku-ku, Tokyo 162-8655 Japan; 12 Department of Viral Pathogenesis and Controls, Research Institute, National Center for Global Health and Medicine, Ichikawa, Chiba 272-8516 Japan; 13 Center for Medical Informatics and Intelligence, National Center for Global Health and Medicine, Shinjuku-ku, Tokyo 162-8655 Japan; 14 National Center for Child Health and Development, Setagaya-ku, Tokyo 157-8535 Japan; 15 Center for Regenerative Medicine, National Center for Child Health and Development, Setagaya-ku, Tokyo 157-8535 Japan; 16 Department of Maternal-Fetal Biology, National Center for Child Health and Development, Setagaya-ku, Tokyo 157-8535 Japan; 17 Department of Genome Medicine, National Center for Child Health and Development, Setagaya-ku, Tokyo 157-8535 Japan; 18 Research Institute, National Center for Geriatrics and Gerontology, Obu, Aichi 474-8511 Japan; 19 Central Biobank, National Center Biobank Network, Shinjuku-ku, Tokyo 162-8655 Japan; 20 Genome Medical Science Project (Toyama), Research Institute, National Center for Global Health and Medicine, Shinjuku-ku, Tokyo 162-8655 Japan

**Keywords:** Next-generation sequencing, Disease genetics

## Abstract

Multiple system atrophy (MSA) is a neurodegenerative disorder characterized by various combinations of autonomic failure, parkinsonism, and cerebellar ataxia. To elucidate variants associated with MSA, we have been conducting short-read-based whole-genome sequence analysis. In the process of the association studies, we initially focused on *GBA1*, a previously proposed susceptibility gene for MSA, to evaluate whether *GBA1* variants can be efficiently identified despite its extraordinarily high homology with its pseudogene, *GBA1LP*. To accomplish this, we conducted a short-read whole-genome sequence analysis with alignment to GRCh38 as well as Sanger sequence analysis and compared the results. We identified five variants with inconsistencies between the two pipelines, of which three variants (p.L483P, p.A495P–p.V499V, p.L483_M489delinsW) were the results of misalignment due to minor alleles in *GBA1P1* registered in GRCh38. The miscalling events in these variants were resolved by alignment to GRCh37 as the reference genome, where the major alleles are registered. In addition, a structural variant was not properly identified either by short-read or by Sanger sequence analyses. Having accomplished correct variant calling, we identified three variants pathogenic for Gaucher disease (p.S310G, p.L483P, and p.L483_M489delinsW). Of these variants, the allele frequency of p.L483P (0.003) in the MSA cases was higher than that (0.0011) in controls. The meta-analysis incorporating a previous report demonstrated a significant association of p.L483P with MSA with an odds ratio of 2.85 (95% CI; 1.05 – 7.76, *p* = 0.0400).

## Introduction

*GBA1* (HGNC:4177) is the gene encoding a lysosomal enzyme, glucocerebrosidase, located on chromosome 1q21. *GBA1* consists of 11 exons spanning 7.6 kb, and a highly homologous pseudogene (*GBA1LP*, HGNC:4178) is located approximately 16 kb apart from the functional *GBA1* gene [1–3]. Unequal pairing with rearrangement between *GBA1* and *GBA1LP* is a frequent cause leading to the generation of gene–pseudogene rearrangements, many of which are causative variants for Gaucher disease. Of these rearrangements, nonreciprocal recombination (gene conversion) events are the most frequent, in which a portion of the functional gene sequence is replaced by the corresponding part of the pseudogene [1, 2, 4].

Biallelic pathogenic variants of *GBA1* cause deficient glucocerebrosidase activities leading to the development of Gaucher disease [4]. Starting from the observation that family members of patients with Gaucher disease have a considerably high prevalence of Parkinson disease (PD) [5], numerous studies have consistently shown that deleterious variants of *GBA1* are strong risk factors for the development of PD [6–9]. Furthermore, we have reported that *GBA1* variants pathogenic for Gaucher disease are also significantly, albeit with a small odds ratio, associated with an increased risk of developing multiple system atrophy (MSA), which is also a neurodegenerative disorder characterized by various combinations of autonomic failure, parkinsonism, and cerebellar ataxia along with the accumulation of α-synuclein within oligodendroglia [10]. Other studies, however, have not demonstrated the associations of *GBA1* variants with MSA, possibly owing to their small sample sizes [11–13].

Given the remarkable progress of short-read sequencing technologies employing next-generation sequencers, comprehensive mutational analysis has become a standard procedure for large-scale studies. Owing to the extraordinarily high homology between *GBA1* and *GBA1LP*, however, short-read sequencing techniques without specific amplification of *GBA1* pose a challenge to the accurate sequence analysis of *GBA1*. Therefore, PCR designed to specifically amplify *GBA1* followed by Sanger sequencing is usually needed for validation of the genotypes in this locus [14].

To investigate the molecular basis of MSA, we have been conducting short-read whole-genome sequence (WGS) analysis of large-scale MSA cases to explore genes associated with MSA. As the initial step for the association studies, we focused on *GBA1*, which we previously reported to be associated with MSA [10]. In this study, we extracted all the variants mapped to *GBA1* as well as those mapped to *GBA1LP* from the WGS data obtained employing short-read sequencers. During the analysis, we noticed a critical pitfall in *GBA1*–*GBA1LP* genotyping that leads to the miscalling of variants based on the short-read sequence data depending on the reference genome. We herein report the details underlying the miscalling events. We also briefly present the results of a potential association of the *GBA1* variants with MSA.

## Materials and methods

### Subjects

Genomic DNA was extracted from peripheral blood leukocytes with written informed consent from 500 patients with MSA registered in the Japan Multiple System Atrophy Registry (Japan MSA registry) (https://msajp.org/) [15] from August 2016 through September 2022. The Japan MSA registry is a multicenter-based prospective cohort study participated by 13 institutions in Japan that enrolls patients with possible or probable MSA on the basis of the revised Gilman criteria [16]. There was no overlap of the MSA cases with those described in our previous study [10]. As the control dataset, variant information data obtained from 9474 healthy individuals or patients with some common diseases were provided from the National Center Biobank Network (NCBN) [17]. Of the control samples, 358 with the registered diagnosis classified into the category of neurodegenerative diseases were not included in the association study. For the association study of *GBA1* variants with MSA, relatedness between samples was checked with KING [18] using the data of the whole genome sequence data described below, and duplicated samples or those with 2nd degree or higher relationships were removed. Ancestry estimation was conducted for all the samples with Somalier using the whole genome sequence data [19] and those with ancestry estimation other than East Asian origin were removed. As the result, 499 MSA cases and 8777 controls were used for the following association study. The research protocol was approved by the institutional review board of each participating institution.

### Sanger sequence analysis of polymerase chain reaction (PCR) products

PCR was conducted employing three primer pairs designed to selectively amplify exons 1–5, 5–7, and 8–11 of *GBA1* but not those of *GBA1LP*, as previously described [6, 10, 20]. Direct nucleotide sequence analysis of the 11 coding regions and the splice sites of *GBA1* was conducted employing a 3730xl Genetic Analyzer (Life Technologies, Carlsbad, CA). The sequences were analyzed using Sequence Scanner (Version 1.0, Applied Biosystems, 2005) and compared with the human *GBA1* sequence using the GRCh38/hg38 Assembly. *GBA1* variants were annotated based on RefSeq NM_00157.4 (NP_000148.2).

### Short-read WGS analysis

WGS analysis was conducted employing the NovaSeq 6000 (Illumina, San Diego, CA) platform with 150-bp paired-end reads with a target depth of 30× for all 500 samples. WGS analysis of the 168 MSA samples was conducted at the International University of Health and Welfare (IUHW). WGS analysis of the 332 MSA samples and the 9116 control samples of the NCBN was conducted at the National Center for Global Health and Medicine (NCGM) [17]. Alignment to GRCh38 and variant calling of all the short reads were conducted employing the Parabricks v3.1.0 (Nvidia, Santa Clara, CA, US), which provides the capability to perform the analysis recommended by GATK at high speed using a GPU [21]. Generated gVCF files were then joint-called using the gVCFtyper program of the Sentieon package [22]. Alignment of the short reads was conducted for both GRCh37 and GRCh38 to compare the results of the variant calling because there are differences in the registered alleles in the *GBA1–GBA1LP* regions between the two reference genomes, as described below.

### Long-read WGS analysis

We additionally conducted a long-read WGS analysis employing PacBio Sequel II (Pacific Biosciences, Menlo Park, CA) to further confirm the accurate variant calling of the *GBA1–GBA1LP* region, for which it was difficult to conclusively explain the inconsistencies between the results obtained by the Sanger and those by the short-read sequence analyses from a case with a structural variant. The HiFi reads were aligned to the reference genome of GRCh38 using Minimap2 [23] with the default parameter settings. The reads aligned to the *GBA1*–*GBA1LP* locus were visualized with IGV and reviewed manually to confirm the structural variation [24].

### Extraction of variants pathogenic for Gaucher disease in patients with MSA

The pathogenicity of *GBA1* variants for Gaucher disease was determined based on whether they were previously reported as pathogenic for Gaucher disease. We referred to the Human Gene Mutation Database (HGMD^®^) Professional 2023.2 to identify previously published reports [25]. The allele frequency of each variant in the MSA samples was compared with that in the control samples obtained from mapping to GRCh37 and our previous report [10], and Fisher’s exact test was conducted using R version 2.8.0.

## Results

In the 500 MSA cases, we identified a total of 13 variants including eight missense (p.I20V, p.R202Q, p.R301H, p.S310G, p.V334I, p.L483P, p.A495P, and p.I528V) variants, two synonymous (p.V499V and p.K505K) variants, and three variants including a splicing variant (c.115+1G>A) and two indels (p.H313del and c.del1447_1466insTG) by either short-read or Sanger sequence analysis (Supplementary Table 1). Notably, inconsistencies in variant calling between the Sanger and the short-read sequence analyses were observed in variant calls for the five variants (p.A495P–p.V499V and c.115+1G>A were called only by short-read sequence analysis, whereas p.H313del, p.L483P and c.del1447_1466insTG were called only by Sanger sequence analysis) (Table 1).

**Table 1:** Variants in *GBA1* with inconsistencies among the Sanger, short-read, and long-read sequence analyses

Case No	Sanger	Short-read (GRCh38)	Short-read (GRCh37)	Long-read	Reason underlying the inconsistency
1	c.1448T>C (p.L483P)^a^	-	c.1448T>C (p.L483P)^a^	Not conducted	Misalignment attributable to the minor alleles registered in *GBA1LP* of GRCh38
2	-	*c.1483G*>*C (p.A495P)*, *c.1497G*>*C (p.V499V)*	-	Not conducted	Misalignment attributable to the minor alleles registered in *GBA1LP* of GRCh38
3	c.del1447_1466insTG^a^, (p.L483_M489delinsW)^a^	-	c.del1447_1466insTG^a^, (p.L483_M489delinsW)^a^	Not conducted	Misalignment attributable to the minor alleles registered in *GBA1LP* of GRCh38
4	*c.937_939del (p.H313del)*	*c.115 +1G*>*A*	*c.115 +1G*>*A*	Recombination in *GBA1LP*^a^, (NC_00001.11:g.155213012_155218391delins [NC_00001.11:g.155233640_155237400;NC_00001.11:g.155237404_155240628])	Large structural variant in the *GBA1LP*

Miscalled variants are described in *Italics*

^a^Confrimed variants

### Misalignment attributable to rare variants registered in *GBA1LP* of the reference genome (GRCh38)

#### Case 1: p.L483P was detected by Sanger sequence analysis, but undetected by short-read sequence analysis with alignment to GRCh38

**Fig. 1:**
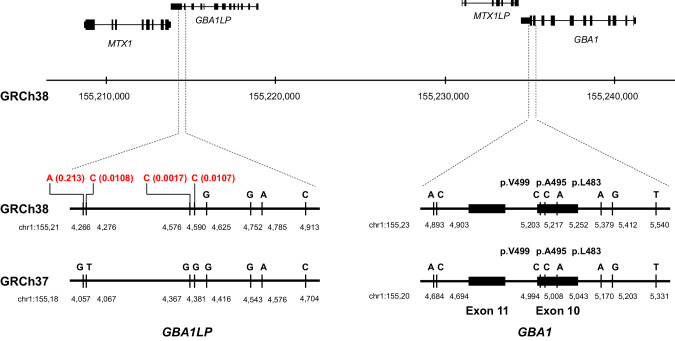
Physical map of *GBA1*, *GBA1LP*, *MTX1*, and *MTX1LP*. The positions of the major alleles different between *GBA1* and *GBA1LP* are shown. Allele frequencies from the Japanese population (8.3KJPN) are displayed in red at the positions on *GBA1LP* (GRCh38) where the minor alleles, identical to the major alleles in *GBA1*, are registered. In GRCh37, the major alleles are registered at the corresponding positions

**Fig. 2:**
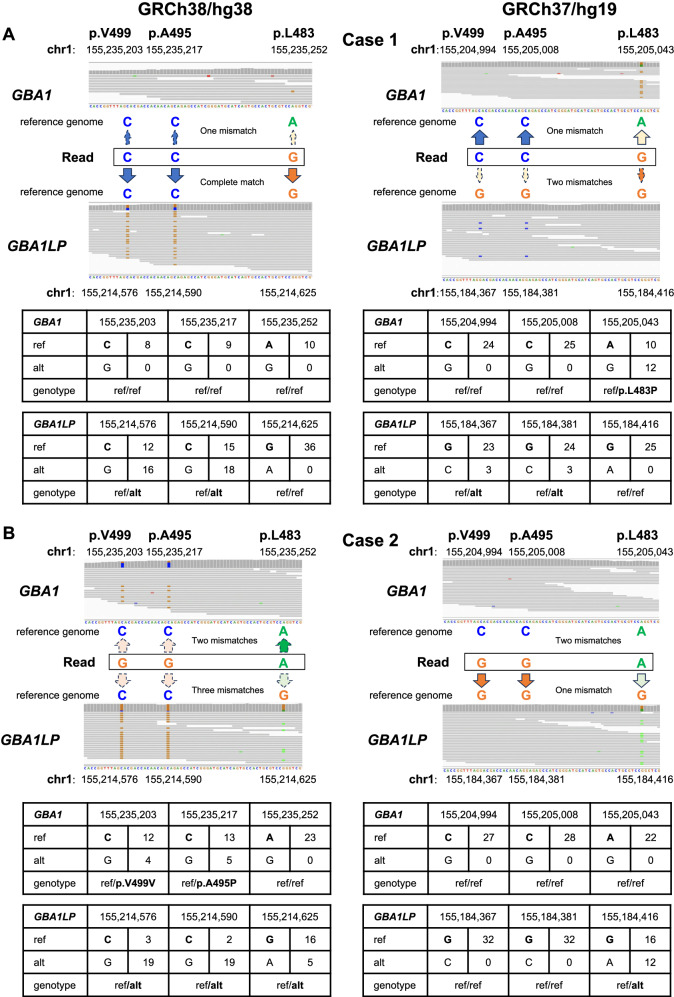
Misalignment of short reads attributable to rare variants registered in *GBA1LP* of the reference genome (GRCh38) in cases 1 and 2. Short-read alignments to *GBA1* and *GBA1LP* in each case are displayed using IGV (with the mapping quality threshold = 20). The tables below the images of IGV show the allele depths retrieved from VCF files at the base positions on chromosome 1 of the reference genomes used for alignment. Note that the number of read bars shown in the IGV based on BAM files does not necessarily completely match with the read depths retrieved from the VCF. **A** Case 1 No variants were called by short-read sequence analysis, whereas p.L483P was detected by Sanger sequence analysis. When the short reads were aligned to GRCh38, the reads with C–C–G showed a complete match at chr1:155,214,576, chr1:155,214,590, and chr1:155,214,625 in *GBA1LP*, whereas the reads showed a mismatch at chr1:155,235,252 in *GBA1*. Consequently, p.L483P was not called a variant in *GBA1*. When the reads were aligned to GRCh37, the reads containing C–C–G showed two mismatches at chr1:155,184,367 and chr1:155,184,381 in *GBA1LP*. Consequently, p.L483P is called a variant in *GBA1*. **B** Case 2 p.A495P and p.V499V were called by short-read sequence analysis, whereas no variants were detected by Sanger sequence analysis. When the short reads were aligned to GRCh38, the reads with G–G–A showed two matches at chr1:155,235,203 and chr1:155,235,217 in *GBA1*, and the reads showed three mismatches at chr1:155,214,576, chr1:155,214,590, and chr1:155,214,625 in *GBA1LP*, resulting in alignment of the reads containing G–G–A to both *GBA1* and *GBA1LP*. Consequently, p.A495P and p.V499V were called variants in *GBA1*. When the reads were aligned to GRCh37, the reads containing G–G–A had two mismatches to the reference genome of *GBA1* at chr1:155,235,203 and chr1:155,235,217, whereas they had only one mismatch to the reference genome of *GBA1LP* at chr1: 155,184,416, and all the reads were correctly aligned to the *GBA1LP* sequences. As a result, neither p.A495P nor p.V499V was called at *GBA1*

Chr1:155,233,639 – chr1:155,235,252 containing exons 10–11 of *GBA1* and adjacent *MTX1LP* have an extraordinarily high sequence homology with the corresponding *GBA1LP* and *MTX1* regions located in the vicinity of *GBA1* on chromosome 1. In *GBA1LP*, GRCh38 registers a minor allele C with an allele frequency (AF) of 0.0017 instead of a major allele G at chr1:155,214,576, a minor allele C with an AF of 0.0107 instead of a major allele G at chr1:155,214,590, a minor allele C with an AF of 0.0108 instead of a major allele T at chr1:155,214,276, and a minor allele A with an AF of 0.213 instead of a major allele G at chr1:155,214,266 (Fig. 1). In contrast, GRCh37 registers major alleles at these four positions in *GBA1LP*. When the short reads were aligned to GRCh38, the reads containing C–C–G completely matched with the reference at chr1:155,214,576, chr1:155,214,590, and chr1:155,214,625 in *GBA1LP*, while the reads showed a mismatch at chr1:155,235,252 in *GBA1*. Consequently, p.L483P was not called a variant in *GBA1* by the short-read sequence analysis (Fig. 2A). When the reads were aligned to GRCh37, the reads containing C–C–G showed two mismatches at chr1:155,184,367 and chr1:155,184,381 in *GBA1LP*, whereas the reads showed only one mismatch at chr1:155,205,043 in *GBA1*, resulting in correct alignment of all the short reads containing A at chr1: 155,205,043 (GRCh37) to the *GBA1* locus. Consequently, p.L483P was called a variant in *GBA1*.

Given the above results, we reanalyzed the variant calling of the other two cases with p.L483P in detail (Cases 5 and 6). In Cases 5 and 6, p.L483P was identified by Sanger sequence analysis as well as by short-read sequence analysis even with alignment to GRCh38. However, the allele depths in the VCF files obtained from alignment to GRCh38 were 13 and 4 (ref/alt) in Case 5, and 15 and 3 (ref/alt) in Case 6, indicating markedly biased allele balances, and reduced reliability in the variant calling. When a filtering option was set based on allele balance or read depth, p.L483P may well be missed in variant calling. In contrast, mapping to GRCh37 resulted in more reliable variant calling, with allele depths of 13 and 15 (ref/alt) and 16 and 7 (ref/alt) in Case 5 and Case 6, respectively.

#### Case 2: p.A495P and p.V499V were called by short-read sequence analysis with alignment to GRCh38, whereas the variants were not detected by Sanger sequence analysis

Similar to Case 1, due to the minor alleles registered in the *GBA1LP* in the reference genome (GRCh38), a misalignment of reads was observed. When the reads were aligned to GRCh38, the reads containing G–G–A showed two mismatches at chr1:155,235,203 and chr1:155,235,217 in *GBA1*, and the reads showed three mismatches at chr1:155,214,576, chr1:155,214,590, and chr1:155,214,625 in *GBA1LP* (Fig. 2B). Thus, the short-read sequence analysis employing GRCh38 as the reference genome could not unequivocally determine whether the reads containing G–G–A are derived from *GBA1* or *GBA1LP*. Indeed, while the majority of the short reads containing G–G–A were aligned to *GBA1LP*, a limited number of short reads containing G–G–A were aligned to *GBA1*. Consequently, p.A495P and p.V499V were called by short-read sequence analysis with alignment to GRCh38. When the reads were aligned to GRCh37 (major alleles are registered in *GBA1LP* at the four positions), the reads containing G–G–A showed two mismatches to the reference genome of *GBA1* at chr1:155,235,203 and chr1:155,235,217, whereas they showed only one mismatch to the reference genome of *GBA1LP*. As a result, reads containing G–G–A were fully aligned to the *GBA1LP* locus, and the p.A495P and p.V499V variants were not called in *GBA1*.

#### Case 3: c.del1447_1466insTG was detected by Sanger sequence analysis, but not called by short-read sequence analysis with alignment to GRCh38

Similar to Cases 1 and 2, the registration of four minor alleles in *GBA1LP* in GRCh38 increases the homology between *GBA1* and *GBA1LP*, leading to fewer reads being specifically aligned to *GBA1*. Consequently, c.del1447_1466insTG was not called by short-read sequence analysis with alignment to GRCh38. When a read does not contain a *GBA1*-specific variant, it could be aligned to either *GBA1* or *GBA1LP*, leading to a MAPQ value of 0. As a result, there are only three reads with a MAPQ > 20 (Supplementary Fig. 1), and the variant was missed through the variant calling process. Conversely, when the reads were aligned to GRCh37, which registers major alleles in *GBA1LP*, it became easier to align the reads specifically to *GBA1*. Consequently, there are twelve reads with a MAPQ > 20, and the variants were accurately called employing GRCh37 as the reference genome through the variant calling process.

### Misalignment attributable to a structural variant involving *GBA1/GBA1LP*

#### Case 4: c.115+1G>A was called by short-read sequence analysis, while p.H313del was called by Sanger sequence analysis

**Fig. 3:**
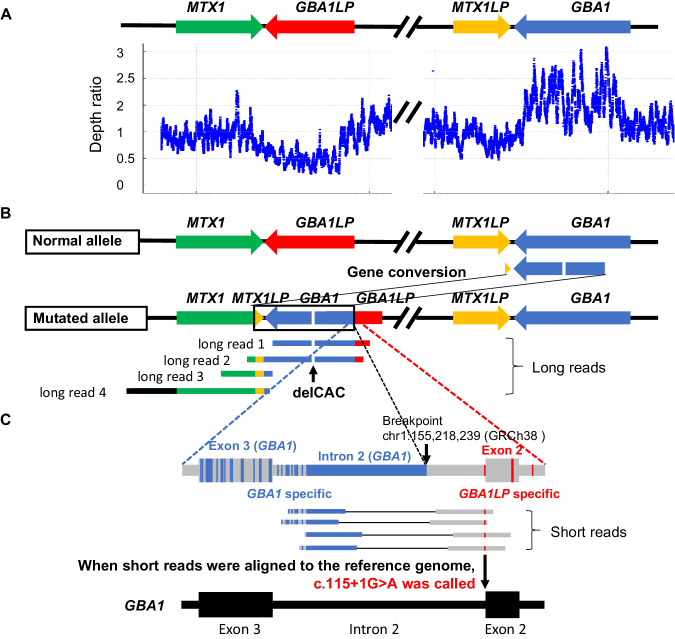
Structural variant involving the region spanning intron 2 of *GBA1LP* and intron 5 of *MTX1*. **A** The read depth ratios of the short reads aligned to *GBA1*, *GBA1LP*, *MTX1*. and *MTX1LP* of Case 4 were calculated as the ratio to the average read depth of the five control subjects, and plotted with chromosomal positions. The depth ratio in the *GBA1* region was increased to about 1.5, while that in the *GBA1LP* region was decreased to about 0.5, raising the possibility that the copy numbers of *GBA1* and *GBA1LP* are three and one, respectively. **B** Physical maps of the *GBA1–GBA1LP* locus in the reference genome and the structural variant derived from gene conversion involving the *GBA1LP*–*MTX1* locus in Case 4 are shown. The region spanning intron 2 of *GBA1LP* (ENST00000689630.1) and intron 5 of *MTX1* was replaced by the region spanning intron 2 of *GBA1* and intron 5 of *MTX1LP* with a deletion of CAC (c.937_939del) in this region (NC_00001.11:g.155237400_155237404), which was clearly demonstrated by the four long-reads 1–4. **C** Across the breakpoint of *GBA1* and *GBA1LP* of the complex structural variant, there are several sets of paired-end reads that were preferentially aligned to *GBA1* because one of the paired-end reads contains an increased number of bases specific to the *GBA1* reference sequence than those specific to *GBA1LP*. Consequently, c.115+1G>A was miscalled at *GBA1* by the short-read sequence analysis. The regions with nucleotide sequences identical between *GBA1* and *GBA1LP* are shown in gray. The regions with nucleotide sequences specific to *GBA1* are shown in blue, whereas those specific to *GBA1LP* are shown in red

When the read depth data of the short-read sequence analysis were analyzed, we noticed that the read depths in the part of the *GBA1* region were increased to about 1.5, whereas those in the *GBA1LP* region were decreased to about 0.5, raising the possibility that the copy numbers of *GBA1* and *GBA1LP* regions are three and one, respectively (Fig. 3A). Of note, long-read sequence analysis revealed that the long-reads 1 and 2 indeed contained a chimeric structure containing the *GBA1LP–MTX1* and the *GBA1* regions. The long-reads 3 and 4 contained a chimeric structure containing the *GBA1LP–MTX1* and the *GBA1–MTX1LP* regions (Fig. 3B). Based on these results, we concluded that a gene conversion event occurred in the *GBA1LP*–*MTX1* region due to the extraordinarily high homology between the *GBA1–MTX1LP* and *GBA1LP–MTX1* regions. The region spanning the intron 2 of *GBA1LP* and the intron 5 of *MTX1* (NC_00001.11:g.155213012_155218391 indicated by a box in Fig. 3B) was replaced by the region spanning the intron 2 of *GBA1* and the intron 5 of *MTX1LP* (NC_00001.11:g.155233640_155240628). Of note, there is a deletion of CAC in this region (NC_00001.11:g.155237401_155237403del). Taken together with these observations, the complex structural variant is described as　NC_00001.11:g.155213012_155218391delins[NC_00001.11:g.155233640_155237400; NC_00001.11:g.155237404_155240628] according to the guidelines of the Human Genome Variation Society (https://www.hgvs.org/content/guidelines). Consistent with the above interpretation, one of the paired-end reads spanning the breakpoint between *GBA1* (upstream) and *GBA1LP* (downstream) contained *GBA1*-specific sequences derived from *GBA1*. On the other hand, the other reads contained sequences derived from *GBA1LP*, whose sequences are identical to that of *GBA1* except for a mismatched base corresponding to c.115+1G>A in *GBA1*. Consequently, the reads were aligned to *GBA1* resulting in the miscalling of c.115+1G>A in *GBA1* (Fig. 3C). Regarding the p.H313del, which was called only by Sanger sequence analysis as the variant in *GBA1*, it was found to be a variant located in the structural variant involving *GBA1LP* as determined by the long-read sequence analysis (Fig. 3B). Thus, both Sanger sequence and short-read sequence analyses failed to correctly call the variants.

### Variants pathogenic for Gaucher disease identified in MSA cases

**Fig. 4:**
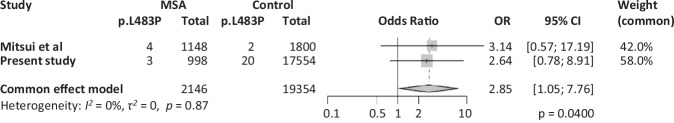
Forest plot showing the results of the meta-analysis of the association of p.L483P with MSA. The forest plot shows the results of the meta-analysis of the association of p.L483P with MSA combining the current study and the previous report [10]. Squares and horizontal lines represent estimated ORs and 95% CIs for individual series. The size of each square represents the statistical weight, the mean of the effect sizes using the inverse variance of the individual studies. Diamonds show the overall effects with 95% CIs. Since between-study variability was not observed (*I*^2^ = 0%, *τ*^2^ = 0, *p* =0.87), the common effects model was employed for the meta-analysis, which demonstrated the odds ratio of 2.85 (95% CI; 1.05 – 7.76, *p* = 0.0400), indicating that the allele frequency of p.L483P is significantly higher in MSA than in control samples in the Japanese population

Pathogenic variants for Gaucher disease identified in the MSA cases include p.S310G, p.L483P, and c.del1447_1466insTG (Table 2). Of the 499 MSA cases, three were heterozygous for p.L483P (AF: 0.003), one was heterozygous for c.del1447_1466insTG (AF: 0.001), and one was heterozygous for p.S310G (AF: 0.001) (Supplementary Table 1). In summary, five of the 499 (AF: 0.005) MSA cases were carriers of *GBA1* variants pathogenic for Gaucher disease. The demographic features of the five MSA patients with the *GBA1* variants are described in Supplementary Table 2. The ages at onset of the five patients ranged from 43 to 73 with the mean age at onset (standard deviation) of 58.6 (9.6), which was not significantly different from those of non-carriers. The clinical phenotypes of these five patients included three MSA-C and two MSA-P patients. Four patients were classified as probable MSA, while one was classified as possible MSA. The combined allele frequency of all the pathogenic variants in the MSA cases was higher than those described in the previous report [10], which, however, did not reach statistical significance. Of note, the allele frequency of p.L483P (0.003) in the MSA cases in this study was comparable to that (0.0035) described in the previous report and higher than that (0.0011) in control samples, which, however, did not reach a statistical significance, either (Table 2). We then conducted a meta-analysis of p.L483P combining the results of the current study (3/998 alleles for the disease group vs 20/17,554 alleles derived from the control group) and those of our previous study (4/1148 alleles for the disease group vs 2/1800 alleles for the control group) [10]. Since between-study variability was not observed (*I*^2^ = 0%, *τ*^2^ = 0, *p* = 0.87), the common effects model was employed for the meta-analysis, which demonstrated the odds ratio of 2.85 (95% CI; 1.05 – 7.76, *p* = 0.0400), indicating that the allele frequency of p.L483P is significantly higher in MSA than in control samples in the Japanese population (Fig. 4)

**Table 2:** Variants pathogenic for Gaucher disease in MSA cases and controls

Variant	Genotype	Present study			Mitsui et al. [10]		
		MSA cases (AC = 998)	Control samples (AC = 17,554)		MSA cases(AC = 1148)	Controls (AC = 1800)	
		Individuals (AF)	Individuals (AF)	*p*-value^a^	Individuals (AF)	Individuals (AF)	*p*-value^b^
p.S310G	Heterozygous	1 (0.00100)	3 (0.000171)	0.198	0	0	-
c.del1447_1466insTG	Heterozygous	1 (0.00100)	0	0.0538	0	0	-
p.L483P	Heterozygous	3 (0.00301)	20 (0.00114)	0.124	4 (0.0035)	2 (0.0011)	0.216

*AC* allele count, *AF* allele frequency

^a^Fisher’s exact test was conducted to compare the allele frequencies in the MSA of this study with those in control samples with mapping to GRCh37

^b^The comparison of allele frequencies reported in our previous study was cited from Mitsui et al. [10]

## Discussion

In this study, we analyzed *GBA1* in a large number of Japanese MSA cases using both Sanger and short-read whole-genome sequence analyses to investigate the potential association of variants pathogenic for Gaucher disease with MSA. In the course of the sequence analysis, we noticed that miscalling could occur in variant calling based on short-read sequence analysis, which is attributable to the minor variants registered in *GBA1LP* in the GRCh38 reference genome but not in the GRCh37 reference genome. In addition, we noticed both Sanger and short-read sequence analyses failed to correctly call a large structural variant involving the *GBA1LP* locus.

As mentioned above, the four minor alleles at chr1:155,214,576, chr1:155,214,590, chr1:155,214,276, and chr1:155,214,266 of GRCh38 reference genome in *GBA1LP*, which indeed correspond to the major alleles of *GBA1*. The presence of these minor alleles in the GRCh37 causes misalignment, wherein short reads derived from *GBA1* are aligned to *GBA1LP* or those derived from *GBA1LP* are aligned to *GBA1*. Specifically, this misalignment caused the disappearance of p.L483P due to the reads derived from *GBA1* being mapped to *GBA1LP*, and the incorrect calling of p.A495P–p.V499V in *GBA1*, which actually originated from reads derived from *GBA1LP*. Although p.L483P was called by the short-read sequence analysis with alignment to GRCh38 in two of the three samples with MSA, these two cases also exhibited variant calling results with reduced confidences (Cases 5 and 6). When a filtering option was set based on allele balance or read depth, p.L483P may well be missed in variant calling using GRCh38 as the reference genome. Since p.L483P is one of the most prevalent pathogenic variants for Gaucher disease worldwide [1, 26] and one with the biggest impact of increasing the risk of PD across different ancestries [27], this issue is critical in clinical sequencing for suspected Gaucher disease and familial as well as sporadic PD.

Similar miscalling of variants is presumably present in the allele frequency databases based on GRCh38. For instance, gnomAD v2.1.1, which is based on alignment to GRCh37, registers p.L483P with an allele frequency of 0.0006703, whereas gnomAD v3.1.2, which is based on alignment to GRCh38, registers p.L483P with an allele frequency of 0.0002369. The lower frequency of p.L483P in v3.1.2 than that in v2.1.1, despite targeting similar ancestry groups, is presumably due to the underestimation of the p.L483P frequency depending on alignment to GRCh38. Additionally, the Japanese allele frequency database, jMorp (8.3KJPN) (https://jmorp.megabank.tohoku.ac.jp/), was based on data obtained with alignment to GRCh37, whereas databases thereafter (up to the latest 54KJPN) are based on data obtained with alignment to GRCh38, suggesting that similar errors in the allele frequency may be present. Therefore, it is necessary to recognize the reference genome used for mapping and carefully interpret the allele frequency database of the *GBA1* p.L483P variant.

In Case 3, it is likely that c.1447_1466delinsTG was not called during the joint calling process due to a low number of reads specifically mapped to exon 9 of *GBA1*. This issue is also attributed to the presence of the four minor alleles registered in the *GBA1LP*. The existence of these minor alleles in *GBAP1* in GRCh38 results in completely identical sequences at chr1:155,233,639 – chr1:155,235,252 (GRCh38) of *GBA1–MTX1LP* and the corresponding regions of *GBA1LP*–*MTX1*. This leads to a significant decrease in MAPQ values and exclusion of reads from variant calling (Supplementary Fig. 1). Therefore, special attention is required in the variant calling of this region containing exons 10–11 of *GBA1*. As described above, the presence of extraordinarily high homologous regions between *GBA1* and *GBA1LP* is a frequent cause of gene–pseudogene rearrangements, many of which are causative variants for Gaucher disease. These variants may easily be missed based on the short-read sequence analysis. To overcome these problematic variant callings of *GBA1*, mapping to GRCh37 or T2T-CHM13/hs1 can prevent misalignment events because major alleles are registered as the reference sequences at these four positions in *GBA1LP*. Furthermore, a software tool, Gauchian, has recently been developed to detect recombinant alleles and single nucleotide variants in the *GBA1* locus [28]. Gauchian is reported to have a more accurate genotyping performance than the BWA-GATK pipeline [28]. However, particularly in exons 9 to 11 of *GBA1*, false positive and false negative calls are not infrequently observed, and mapping to GRCh37 results in fewer errors than GRCh38, indicating that the presence of the four minor alleles registered in GRCh38 may contribute to some errors even with Gauchian [29]. Furthermore, recombinant alleles of *GBA1* are mistakenly called a copy number change in the *GBA1*–*GBA1LP* locus in some cases, hence results obtained employing Gauchian should be interpreted with caution [29]. When comprehensive variant calling of *GBA1* is pursued, alternative sequencing pipelines including Sanger or long-read sequence analyses would be required for accurate variant calling of these regions.

We also identified that a large structural variant involving the *GBA1–GBA1LP* locus can be misinterpreted as a single nucleotide variant within the *GBA1* locus based on the short-read sequence analysis as well as on the Sanger sequence analysis. The splice donor site variant of c.115+1G>A, miscalled using short-read sequence analysis in Case 4, is known to be a pathogenic variant for Gaucher disease and associated with an increased risk of developing PD in the Ashkenazi Jewish population via exon 2 skipping of glucocerebrosidase mRNA [8, 9, 30]. Furthermore, a previous study showed that the frequency of structural variations in the *GBA1–GBA1LP* locus is considerably high [28]. As such, long-read sequence analysis is preferred for accurate variant calling and should be considered if a large structural variant is suspected when a biased distribution of read depths is observed with short-read sequence data.

Our present study showed that the allele frequency of p.L483P is higher than that in the control samples, which, however, did not reach statistical significance (Table 2). This may be due to a type II error, that is because allele counts of 9948 may be required in each group to reach a statistical significance for a variant with an allele frequency of 0.0030 vs 0.0011 with 80% power and alpha of 0.05. Thus, sufficiently large sample sizes would be required to demonstrate associations of rare variants with MSA. For this reason, we conducted a meta-analysis of p.L483P combining the independent datasets of the current study and those of our previous study [10], which indeed showed a significant association of p.L483P with MSA (Fig. 4). It has been proposed that *GBA1* variants might contribute to the accumulation of α-synuclein in patients with PD [31, 32], while the role of *GBA* variants in the pathogenesis of MSA, which is also an α-synucleinopathy, remains unclear. Since we have recently reported siblings with MSA-C and PD sharing a *GBA1* variant pathogenic for Gaucher disease, the observation may suggest a role of *GBA1* variants as a common genetic basis underlying PD and MSA [33].

In conclusion, given the extraordinarily high homology between *GBA1* and *GBA1LP*, variant calling should be interpreted with caution because different results may be obtained depending on the analysis pipelines. We emphasize the importance of alignment to the appropriate reference genome and utilizing long-read sequencing technology, particularly for this gene locus.

## Supplementary information

Supplementary Figure

Supplementary Table
