# Correction: Association study of *GBA1* variants with MSA based on comprehensive sequence analysis -Pitfalls in short-read sequence analysis depending on the human reference genome-

**DOI:** 10.1038/s10038-024-01293-y

**Published:** 2024-09-20

**Authors:** Kenta Orimo, Jun Mitsui, Takashi Matsukawa, Masaki Tanaka, Junko Nomoto, Hiroyuki Ishiura, Yosuke Omae, Yosuke Kawai, Katsushi Tokunaga, NCBN Controls WGS Consortium, Hatsue Ishibashi-Ueda, Tsutomu Tomita, Michio Noguchi, Ayako Takahashi, Yu-ichi Goto, Sumiko Yoshida, Kotaro Hattori, Ryo Matsumura, Aritoshi Iida, Yutaka Maruoka, Hiroyuki Gatanaga, Akihiko Shimomura, Masaya Sugiyama, Satoshi Suzuki, Kengo Miyo, Yoichi Matsubara, Akihiro Umezawa, Kenichiro Hata, Tadashi Kaname, Kouichi Ozaki, Haruhiko Tokuda, Hiroshi Watanabe, Shumpei Niida, Eisei Noiri, Koji Kitajima, Yosuke Omae, Reiko Miyahara, Hideyuki Shimanuki, Yosuke Kawai, Katsushi Tokunaga, Tatsushi Toda, Shoji Tsuji

**Affiliations:** 1 Department of Neurology, Graduate School of Medicine, The University of Tokyo, 7-3-1 Hongo, Bunkyo-ku, Tokyo 113-8655 Japan; 2 Department of Precision Medicine Neurology, Graduate School of Medicine, The University of Tokyo, 7-3-1 Hongo, Bunkyo-ku, Tokyo 113-8655 Japan; 3 Institute of Medical Genomics, International University of Health and Welfare, 4-3, Kozunomori, Narita-shi, Chiba, 286-8686 Japan; 4 Department of Neurology, Okayama University Graduate School of Medicine, Dentistry and Pharmaceutical Sciences, 2-5-1, Shikata-cho, Kita-ku, Okayama, 700-8558 Japan; 5 Genome Medical Science Project, National Center for Global Health and Medicine, 1-21-1, Toyama, Shinjuku-ku, Tokyo 162-8655 Japan; 6 NCVC Biobank, National Cerebral and Cardiovascular Center, Suita, Osaka, 564-8565 Japan; 7 Medical Genome Center, National Center of Neurology and Psychiatry, Kodaira, Tokyo 187-8551 Japan; 8 Department of Bioresources, Medical Genome Center, National Center of Neurology and Psychiatry, Kodaira, Tokyo 187-8551 Japan; 9 Department of Clinical Genome Analysis, Medical Genome Center, National Center of Neurology and Psychiatry, Kodaira, Tokyo 187-8551 Japan; 10 NCGM Biobank, National Center for Global Health and Medicine, Shinjuku-ku, Tokyo 162-8655 Japan; 11 AIDS Clinical Center, National Center for Global Health and Medicine, Shinjuku-ku, Tokyo 162-8655 Japan; 12 Department of Viral Pathogenesis and Controls, Research Institute, National Center for Global Health and Medicine, Ichikawa, Chiba 272-8516 Japan; 13 Center for Medical Informatics and Intelligence, National Center for Global Health and Medicine, Shinjuku-ku, Tokyo 162-8655 Japan; 14 National Center for Child Health and Development, Setagaya-ku, Tokyo 157-8535 Japan; 15 Center for Regenerative Medicine, National Center for Child Health and Development, Setagaya-ku, Tokyo 157-8535 Japan; 16 Department of Maternal-Fetal Biology, National Center for Child Health and Development, Setagaya-ku, Tokyo 157-8535 Japan; 17 Department of Genome Medicine, National Center for Child Health and Development, Setagaya-ku, Tokyo 157-8535 Japan; 18 Research Institute, National Center for Geriatrics and Gerontology, Obu, Aichi 474-8511 Japan; 19 Central Biobank, National Center Biobank Network, Shinjuku-ku, Tokyo 162-8655 Japan; 20 Genome Medical Science Project (Toyama), Research Institute, National Center for Global Health and Medicine, Shinjuku-ku, Tokyo 162-8655 Japan

**Keywords:** Next-generation sequencing, Disease genetics

Correction to: *Journal of Human Genetics* 10.1038/s10038-024-01266-1, published online 18 July 2024

In this article, the authors conducted a meta-analysis referring to their previous report (Mitsui J, Matsukawa T, Sasaki H, Yabe I, Matsushima M, Dürr A, et al. Variants associated with Gaucher disease in multiple system atrophy. Ann Clin Transl Neurol. 2015;2:417–26. 10.1002/acn3.185). In Table 2 that referred to this report, correct citations are MSA cases (AC = 1148) instead of MSA cases (AC = 1092) and p-value of 0.216 instead of 0.25. In the meta-analysis, 1092 was mistakenly used as the total number of alleles in MSA cases instead of 1148, the correct number. Consequently, the correct results of the meta-analysis are slightly different from those described in the original manuscript. However, the conclusions of the study regarding the association of the *GBA1* variant with MSA remain the same. The following corrections have been made to the article:

In the abstract, “The meta-analysis incorporating a previous report demonstrated a significant association of p.L483P with MSA with an odds ratio of 2.92 (95% CI; 1.08 – 7.90, *p* = 0.0353).” should read “The meta-analysis incorporating a previous report demonstrated a significant association of p.L483P with MSA with an odds ratio of 2.85 (95% CI; 1.05 – 7.76, *p* = 0.0400).”

Figure 4 has been updated as shown below.

**Figure:**
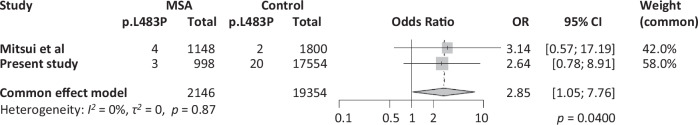

In the Results section, “Since between-study variability was not observed (*I*^2^ = 0%, *τ*^2^ = 0, *p* = 0.83), the common effects model was employed for the meta-analysis, which demonstrated the odds ratio of 2.92 (95% CI; 1.08 – 7.90, *p* = 0.0353)…” should read “Since between-study variability was not observed (*I*^2^ = 0%, *τ*^2^ = 0, *p* = 0.87), the common effects model was employed for the meta-analysis, which demonstrated the odds ratio of 2.85 (95% CI; 1.05 – 7.76, *p* = 0.0400)…”

The original article has been updated.

