# Supplementary material for: Association study of GBA1 variants with MSA based on comprehensive sequence analysis -Pitfalls in short-read sequence analysis depending on the human reference genome-
Source: J Hum Genet. 2024 Jul 18;69(12):613–21. doi: 10.1038/s10038-024-01266-1 (PMC11599039; doi:10.1038/s10038-024-01266-1)
Supplement: Supplementary file 1 — Supplementary Figure [file 10038_2024_1266_MOESM1_ESM.docx]

**
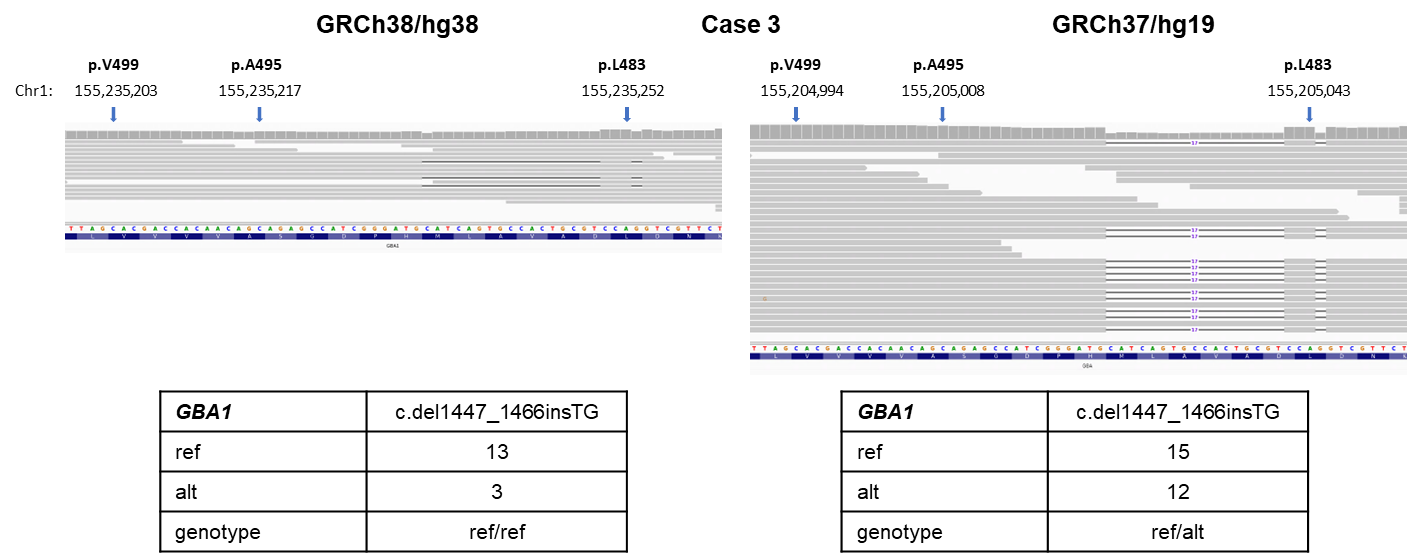
**

**Supplementary Fig. 1 Alignments of short reads to *GBA1* in case 3.**

Short-read alignments to *GBA1* in case 3 is displayed using IGV (with the mapping quality threshold = 20). The tables below the images of IGV show the allele depth located at the indel retrieved from BAM file at the base positions on chromosome 1 of the reference genomes used for alignment.

**
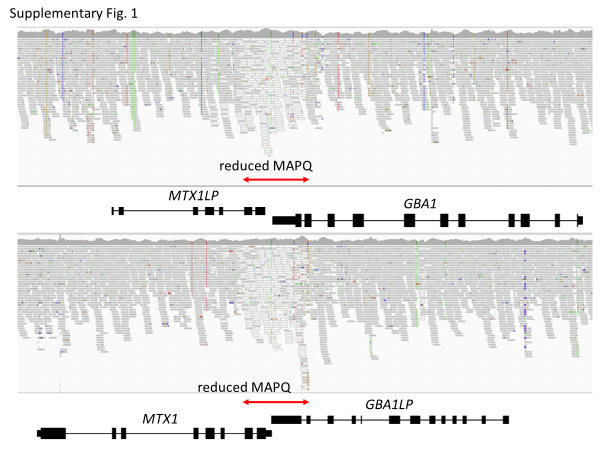
**

**Supplementary Fig. 2 Short reads aligned to the *GBA1*–*MTX1LP* and the *GBA1LP*–*MTX1* regions of GRCh38.**

The short reads of Case 3 aligned to the *GBA1–MTX1LP* and the *GBA1LP–MTX1* regions of GRCh38 are shown employing IGV (with the mapping quality threshold = 0). The reads with MAPQ = 0 are shown by the bars with white color. The chr1:155,233,639 – chr1:155,235,252 (GRCh38) of *GBA1*–*MTX1LP* and chr1:155,213,012 – chr1:155,214,625 (GRCh38) regions of *GBA1LP*–*MTX1* have extraordinarily high identities, resulting in dramatic decrease of MAPQ values.
