# Supplementary material for: Association study of GBA1 variants with MSA based on comprehensive sequence analysis -Pitfalls in short-read sequence analysis depending on the human reference genome-
Source: J Hum Genet. 2024 Jul 18;69(12):613–21. doi: 10.1038/s10038-024-01266-1 (PMC11599039; doi:10.1038/s10038-024-01266-1)
Supplement: Supplementary file 2 — Supplementary Table [file 10038_2024_1266_MOESM2_ESM.docx]

| cDNA  (NP_000148.2) | Protein | Genotype | Number of cases detected in each pipeline (N = 500) | | | Allele frequencies in the control samples |
| --- | --- | --- | --- | --- | --- | --- |
|  |  |  | Sanger | WGS (GRCh38) | WGS (GRCh37) | WGS  (GRCh37) |
| c.58A>G | p.I20V | Homozygous | 3 | 3 | 3 | 0.0662 |
| c.58A>G | p.I20V | Heterozygous | 49 | 49 | 49 | 0.0662 |
| c.115+1G>A | - | Heterozygous | 0 | 1 | 1 | 0.0000570 |
| c.605G>A | p.R202Q | Heterozygous | 2 | 2 | 2 | 0.00296 |
| c.902G>A | p.R301H | Heterozygous | 1 | 1 | 1 | 0.000285 |
| c.928A>G | p.S310G | Heterozygous | 1 | 1 | 1 | 0.000171 |
| **c.937_939del** | **p.H313del** | **Heterozygous** | **1** | **0** | **0** | **-** |
| c.1000G>A | p.V334I | Heterozygous | 1 | 1 | 1 | 0.000171 |
| **c.1447_1466**  **delinsTG** | **p.L483_M489**  **delinsW** | **Heterozygous** | **1** | **0** | **1** | **-** |
| **c.1448T>C** | **p.L483P** | **Heterozygous** | **3** | **2** | **3** | **0.00114** |
| **c.1483G>C** | **p.A495P** | **Heterozygous** | **0** | **1** | **0** | **0.000114** |
| **c.1497G>C** | **p.V499V** | **Heterozygous** | **0** | **1** | **0** | **0.000513** |
| c.1515G>A | p.K505K | Heterozygous | 5 | 5 | 5 | 0.00644 |
| c.1582A>G | p.I528V | Heterozygous | 4 | 4 | 4 | 0.00655 |

**Supplementary table 1** **Variants detected by Sanger and short-read sequence analyses**

Variants with mismatches between Sanger and WGS are indicated in bold.

**Supplementary table 2** **Demographic features of patients MSA patients with *GBA* variants pathogenic for Gaucher disease**

| No | Variant | Age at onset | Sex | Subtype | Diagnosis |
| --- | --- | --- | --- | --- | --- |
| 1 | p.S310G | 43 | Male | MSA-C | probable |
| 2 | p.L483P | 56 | Male | MSA-C | probable |
| 3 | p.L483P | 73 | Female | MSA-C | probable |
| 4 | p.L483P | 61 | Male | MSA-P | probable |
| 5 | p.L483_M489delinsW | 60 | Male | MSA-P | possible |
